# *Penicillium marneffei* Infection in AIDS

**DOI:** 10.4061/2011/764293

**Published:** 2011-02-10

**Authors:** Stephenie Y. N. Wong, K. F. Wong

**Affiliations:** Department of Pathology, Queen Elizabeth Hospital, 30 Gascoigne Road, Kowloon, Hong Kong

## Abstract

*Penicillium marneffei* is a dimorphic fungus which is endemic in Southeast Asia. It is an opportunistic pathogen which has emerged to become an AIDS-defining illness in the endemic areas. Early diagnosis with prompt initiation of treatment is crucial for its management. Prompt diagnosis can often be established through careful cytological and histological examination of clinical specimens although microbiological culture remains the gold standard for its diagnosis. Standard antifungal treatment for AIDS patients with penicilliosis is well established. Highly active antiretroviral therapy should be started early together with the antifungal treatment. Special attention should be paid to potential drug interaction between antiretroviral and antifungal treatments. Secondary prophylaxis may be discontinued with a low risk of relapse of the infection once the immune dysfunction has improved.

## 1. Introduction

*Penicillium marneffei* was first discovered in 1959 by G. Segretain at the Pasteur Institute in Paris. The strain was isolated from bamboo rats dying of disseminated mycosis in Vietnam. The new species was named *P. marneffei* in honour of Hubert Marneffe, the Director of Pasteur Institute in Indochina [1, 2]. The first report of human infection due to *P. marneffei* was also reported by G. Segretain who accidentally pricked his finger with a needle containing the yeast cells of *P. marneffei*. A small nodule appeared at the site of infection followed by lymphangitis 9 days after the accident [3]. The first natural human infection was reported in 1973 from a patient with Hodgkin lymphoma who lived in Southeast Asia [4]. Before the first case was reported in 1988 in a patient infected with the human immunodeficiency virus (HIV) [5], human penicilliosis was uncommon with less than 40 cases reported in the Southeast Asia [6, 7]. However, the incidence of penicilliosis increased rapidly thereafter with the development of HIV pandemic and the infection became one of the commonest acquired immune deficiency syndrome (AIDS)-defining illnesses among HIV-positive patients in endemic areas [8–10]. 

## 2. Mycology

**Figure 1:**
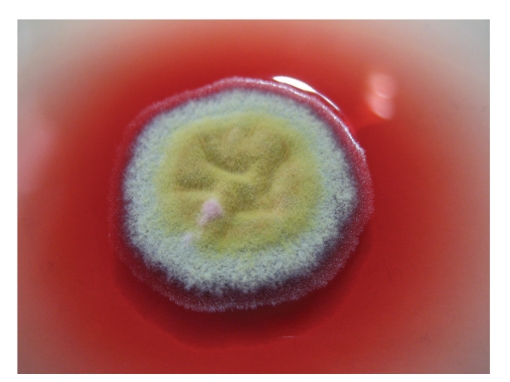
Granular colony of *P. marneffei* with a characteristic red diffusible pigment on Sabouraud's dextrose agar after 7 days incubation at 25°C.

**Figure 2:**
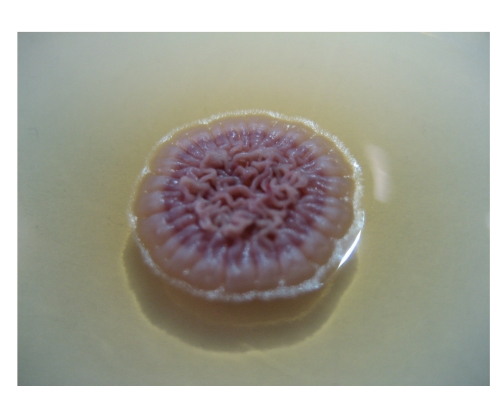
Yeast-like colony of *P. marneffei* without red diffusible pigment on Sabouranud's dextrose agar after 7 days of incubation at 35°C.

**Figure 3:**
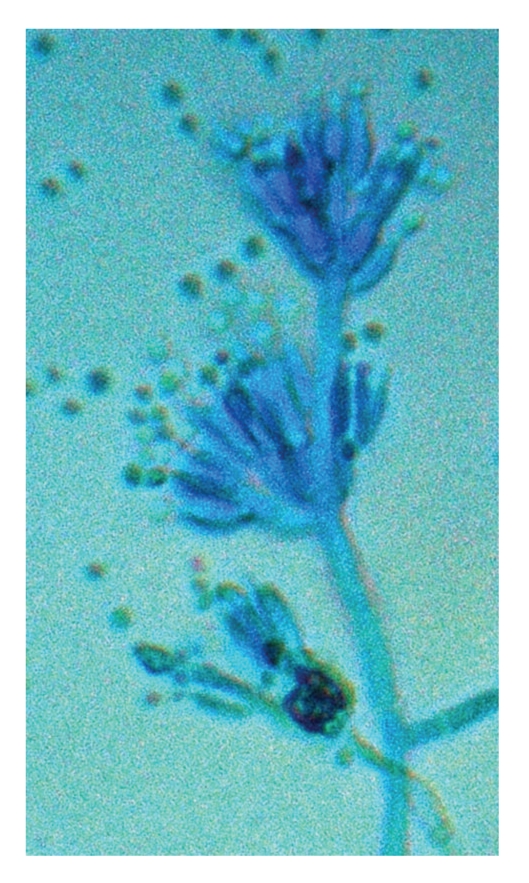
Microscopy of the mold form of *P. marneffei* showing septated hyaline hypae and fruiting structures composing of branching metulae and philiades with spherical condidia in chains (lactophenol cotton blue ×400).

*P. marneffei* is the only dimorphic fungus in the genus of *Penicillium*. It exists in mycelial form at 25°C but yeast form at 37°C [1]. It shows a rapid growth rate and matures within 3 days at 25–30°C. Its growth is enhanced in Sabouraud dextrose agar but is inhibited by cycloheximide [11]. At 25°C, the colonies of *P. marneffei* are granular with shade of greenish-yellow colour and a characteristic red diffusible pigment (Figure 1). Little or no red diffusible pigment is produced at 35 to 37°C (Figure 2). Microscopically, the mold form is typical of other *Penicillium* species with hyaline septated hyphae and fruiting structures composing of branching metulae and phialides which produce spherical conidia in chains (Figure 3).

## 3. Epidemiology

### 3.1. Endemicity

*P. marneffei* infection is endemic among HIV-positive patients in many areas in Southeast Asia, including Thailand, Vietnam, Hong Kong, Southern China, Taiwan, India, and Laos [6, 8, 9, 12–20]. So far, all reported cases of *P. marneffei* infections in AIDS patients have showed epidemiological link with the endemic areas except for one case reported in an African from Ghana who had never been to Southeast Asia [21]. Among the endemic areas, the greatest number of cases was reported in Northern Thailand, where penicilliosis is the third commonest AIDS-indicating disease among HIV-positive patients [8, 22, 23].

### 3.2. Natural Reservoir and Mode of Transmission

A lot is still unknown about the natural reservoir and route of transmission of *P. marneffei*. Human and bamboo rats are the only known animal hosts of *P. marneffei*. Four species of bamboo rats, *Rhizomys sinensis*, *Rhizomys pruinosus*, *Rhizomys sumatrensis* and *Cannomys badius*, are known to be enzootic reservoirs. The distribution of these bamboo rat species generally follows the distribution of endemicity of *P. marneffei* [6, 24–28]. 

It is not certain whether human infection is a result of exposure to infected animals or both bamboo rats and human get infected because of exposure to a common environmental source. The available information seems to suggest the latter. A case-control study in Northern Thailand comparing 80 cases of penicilliosis in patients with AIDS and 160 control patients with AIDS but without penicilliosis showed that exposure or consumption of bamboo rats was not a risk factor for *P. marneffei* infection. On the other hand, a recent history of occupational or other exposure to soil especially during rainy season was found to be a risk factor [29]. An airborne route of transmission through inhalation of conidia from an environmental source with subsequent dissemination to other body sites during immunosuppression has been postulated [13, 30, 31]. However, soil samples obtained from bamboo rat burrows and residential area of patients with penicilliosis were rarely positive for *P. marneffei* [25].

Penicilliosis was reported as a cause of laboratory-associated infection. As demonstrated by G. Segretain, localized infection was possible through direct inoculation of the fungus into the skin [1]. Another laboratory-acquired infection was reported in an undiagnosed HIV-positive physician who visited a laboratory where students were handling *P. marneffei* cultures on the open bench. He developed disseminated infection shortly after the exposure and the presumptive route of acquisition was inhalation [32]. The CDC has recommended Biosafety Level-2 (BSL-2) practices with containment equipment and facilities for propagating and manipulating *P. marneffei* cultures [33].

### 3.3. Incubation Period

The incubation period of *P. marneffei* infection has not been well defined. A report of a patient who lived in an nonendemic area but developed penicilliosis 11 years after visiting Hong Kong has suggested the possibility of a long latency with subsequent reactivation [34]. There is also evidence that primary infection might occur as *P. marneffei *infection can present early in young children who had acquired HIV perinatally [35].

### 3.4. Seasonality

A seasonal pattern of *P. marneffei* infection has been observed in Northern Thailand with increased incidence during the rainy seasons [29, 36]. As there should not be any seasonal variation in the degree of immunosuppression in HIV, the marked seasonality suggests that many of the infections are primary infection and that the heavy rainfall provides a favorable condition for the growth of the fungus, thus increasing the chance of exposure to susceptible host [36].

## 4. Pathology

The pathology of penicilliosis in different organs varies depending on the host immunity. Anergic and necrotizing tissue reaction are often observed in AIDS patients. Granuloma formation will help localize the infection and prevent further dissemination. Failure of this response in AIDS patients may explain the higher rate of disseminated disease [7–9, 13, 37]. 

The most frequent sites of involvement are liver and lungs but lymph node, bone marrow, skin and intestines are also affected. In the liver, histiocyte infiltration of the sinusoids and parenchyma is seen, and epithelioid granuloma may be found. Of interest, no correlation of the liver function test results with the histological changes has been observed [38]. In the lymph node, there is often lymphoid depletion with histiocytic proliferation and focal necrosis [39]. In the bone marrow, histiocytic proliferation can be prominent or subtle, with or without granuloma formation. Rarely, a histiocytic response is lacking [40]. Haemophagocytic syndrome has also been reported [41].

## 5. Clinical Feature

**Figure 4:**
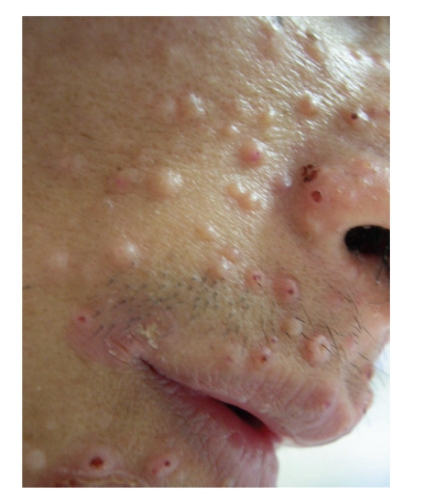
Molluscum-contagiosum-like skin lesions associated with *P. marneffei* infection.

**Figure 5:**
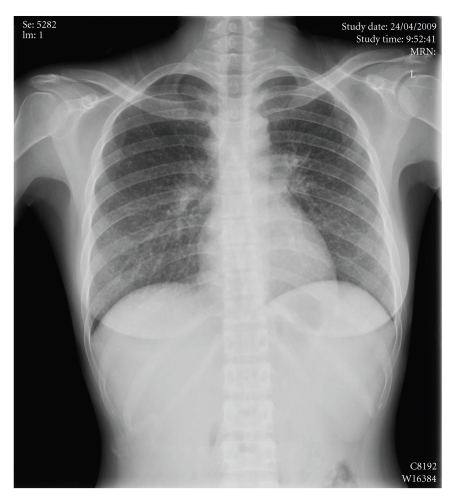
Chest X-ray showing diffuse mottling of both lungs simulating military tuberculosis.

Penicilliosis is mostly seen in late HIV infection with CD4+ count less than 100/uL. Up to 80% or more of the cases have CD4+ count below 50/uL [8, 18, 42].  Table 1 summarizes the clinical features at presentation [8, 17, 18]. Most patients have constitutional symptoms with fever, weight loss and malaise. Skin manifestation such as subcutaneous abscesses and papule-like ulcers may be present [43]. Molluscum-contagiosum-like lesion is not infrequent (Figure 4). It is common to have signs and symptoms reflecting involvement of reticuloendothelial system including anaemia, hepatosplenomegaly and lympadenopathy. Respiratory involvement is often present, with productive cough, dyspnoea and haemoptyisis. Chest X-ray may show diffuse reticular infiltration (Figure 5), localized alveolar infiltrates or cavitary lesion [44]. Diarrhoea is not uncommon and sometimes may be bloody. The infection may rarely present as acute abdomen [45, 46]. Other presenting symptoms include osteoarthritis, genital ulcers and oral lesions [16, 47–51].

Central nervous system involvement is uncommon. A group from Vietnam has, however, reported the development of a syndrome of acute altered mental status with confusion, agitation, or depressed consciousness in the setting of subacute febrile illness [50]. Examination of the cerebrospinal fluid (CSF) could be normal, and abnormal cell count was seen only in one third of the cases. 71% had elevated CSF protein and 24% cases had a CSF glucose/serum glucose ratio <0.5. The disease course was rapidly progressive with a high mortality.

Since penicilliosis is usually seen in advanced stage of HIV infection, 55 to 77% of cases may have other concurrent opportunistic infections such as tuberculosis, disseminated herpes zoster, *Pneumocystis jiroveci* pneumonia, cryptococcosis, toxoplasmosis and should be watched out for [8, 17, 18].

## 6. Laboratory Diagnosis

### 6.1. Cytological and Histological Examination

**Figure 6:**
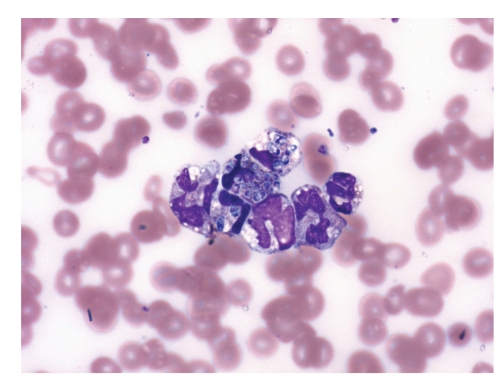
Peripheral blood monocytes with ingested yeast cells (May Grünwald Giemsa ×1000).

**Figure 7:**
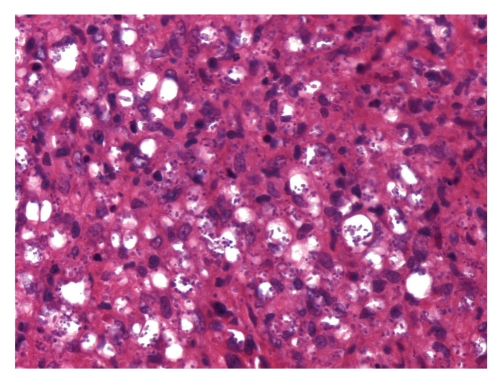
Lymph node biopsy showing histiocytic proliferation with numerous round to oblong yeast cells (haematoxylin and eosin ×400).

**Figure 8:**
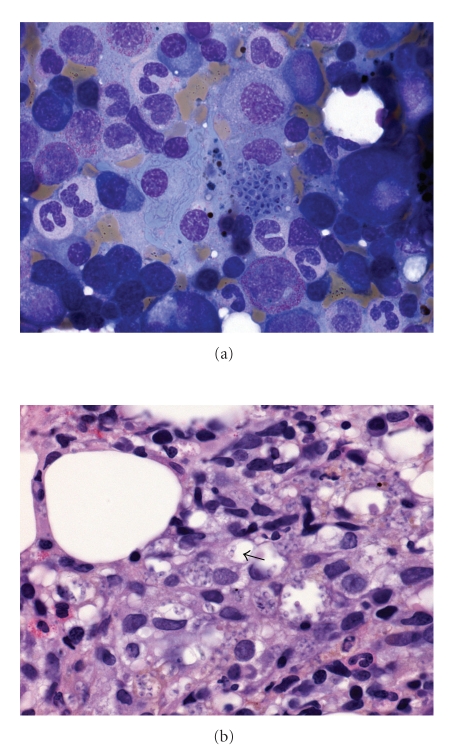
(a) Marrow aspirate showing a histiocyte engorged with yeast cells with reddish pink inclusions (May Grünwald Giemsa ×1000). (b) Trephine biopsy showing histiocytic proliferation with vague granuloma formation and ingested yeast cells. Some yeast cells have transverse septum (arrow) (haematoxylin and eosin ×400).

**Figure 9:**
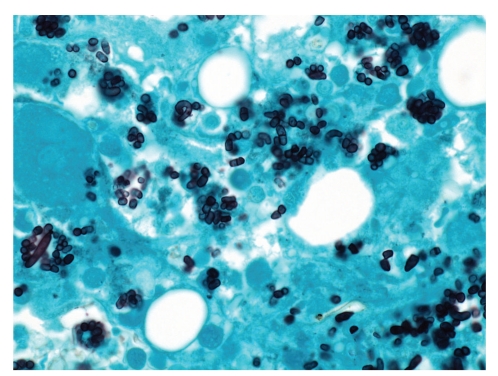
Silver methenamine stain showing colonies of *P. marneffei*. Some yeast cells are oval to oblong in shape with a transverse septum (silver methenamine ×1000).

The diagnosis of penicilliosis may be suspected or made through examination of cytology or biopsy specimens. Cytology specimens are more readily obtained by less invasive procedures such as fine-needle aspiration of lymph nodes, sputum cytology and touch smear of skin [8, 37, 52–54]. For high grade fungaemia, yeast cells may be seen inside monocytes in peripheral blood smear (Figure 6) [40]. The yeast cells may be sparse or abundantly found in histiocytes or extracellularly (Figures 7 and 8), and are most readily demonstrated by fungal stains such as periodic acid-Schiff and silver methenamine stains (Figure 9). Detection of nonbudding yeast cells with characteristic central transverse septum would give a presumptive diagnosis which should be confirmed by microbiological culture.

*P. marneffei* infection can sometimes be histologically occult, and the yeast cells may resemble cellular debris because of their size and staining pattern. Furthermore, granuloma formation may be absent because of anergic response in AIDS. Therefore, fungal stains such as silver methenamine stain should be performed on trephine biopsies in febrile AIDS patients from endemic area even in the absence of marrow granuloma [40].

A number of microorganisms have to be differentiated from *P. marneffei* on cytologic preparation or tissue section. Their distinguishing features are shown in Table 2. *Histoplasma capsulatum* is the commonest microorganism that may be confused with *P. marneffei* due to their similar size and staining properties. Distinction between them relies on the detection of central transverse septum which is characteristic of *P. marneffei* as it reproduced by binary fission or the demonstration of budding yeast cells which are typical of *Histoplasma species*. Epidemiologic link to area of endemicity of the two fungi can also aid in the diagnosis [40, 52, 53, 55].

### 6.2. Microbiological Culture

**Figure 10:**
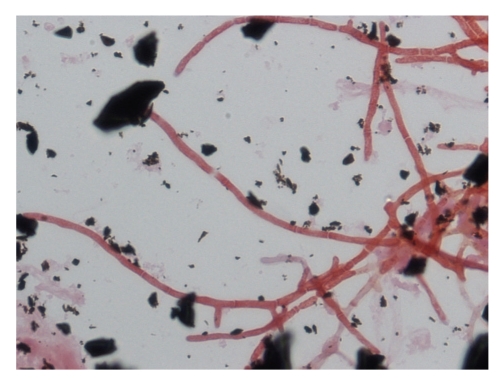
Septated hyphae-like structures but not yeast forms are demonstrated in the initial gram smear taken from a positive blood culture (Gram ×1000).

Isolation of *P. marneffei* remains the gold standard for diagnosis. Among all the clinical specimens studied, the bone marrow gives the highest yield for culture, approaching 100%. This is followed by skin biopsy (90%) and blood culture (76%) [8]. HIV-positive patients with penicilliosis have a higher incidence of fungaemia when compared with HIV-negative patients [58, 59]. Both automated blood culture system and blood culture medium for mycobacterium tuberculosis are able to support the growth of *P. marneffei* [60]. The time to positivity for automated blood culture is around 4 days (range: 1.5–7 days) (personal observation). Although *P. marneffei* exists in yeast form at 37°C, septated hyphae-like structures but not yeast cells are detected in the initial gram smear taken from the positive blood culture (Figure 10). The hyphae structures will break down into arthroconidia-like yeast cells with time.

### 6.3. Serology and Antigen Testing

Various types of antigen and antibody testing specific to *P. marneffei* have been described but they are not widely available [58, 61–67]. It is noted that HIV-positive patients with penicilliosis have a lower level of antibody and a higher level of antigen of *P. marneffei* when compared with HIV-negative patients penicilliosis [58]. Galactomannan assays for *Aspergillus* species is also known to detect the galactomannan of *Penicillium* species and can aid in the diagnosis of penicilliosis. Among 15 cases of penicilliosis in HIV patient, 73.3% was found to be positive with Platelia Aspergillus enzyme immunoassay kit (Bio-rad) with a median OD index of 4.419 [68]. In another series, almost 80% of penicilliosis patient was also found to be galactomannan positive by Pastorex Aspergillus testing (Bio-rad) with a median titre of 1 : 8 [18]. It is now our routine practice to screen all newly diagnosed HIV-patients with galactomannan testing for early detection of potential cases of penicilliosis.

### 6.4. Molecular Testing

PCR assay specific for *P. marneffei* has been developed in research setting but is not available for routine clinical use [69–73].

## 7. Treatment

### 7.1. Antifungal Susceptibility

There is no standardized technique or interpretation criteria for antifungal susceptibility testing for dimorphic fungus. The result of susceptibility testing in dimorphic fungus is influenced by the method, incubation duration, incubation condition and medium used. The inhibitory level of the same drug can be different against the yeast or the mycelial form of the same fungal isolate and the correlation between *in vitro testing* and *in vivo* efficacy is largely unknown [74–76].

*P. marneffei* is susceptible to 5-flucytosine and the azole group of antifungal agents including miconazole, ketoconazole and itraconazole. Fluconazole is the least active among the azoles in *in vitro* setting. Treatment response of the azoles appears to correlate well with *in vitro* result, being high with itraconazole but poor with fluconazole. Amphotericin B is clinically effective although *in vitro* susceptibility test often shows variable results [4, 74, 77].

For the newer antifungal agents, voriconazole has been shown to have activity comparable with that of itraconazole and the preliminary clinical data is encouraging [78, 79]. For the echinocandins, both anidulafungin and micafungin have some degree of activity against *P. marneffei* [76, 80]. In *in vitro* testing, micafungin was found to have synergistic effect with itraconazole and to a lesser degree with amphotericin B against *P. marneffei* [81]. However, it is still uncertain whether this can be translated to clinical management of human infection.

### 7.2. Antifungal Treatment

The mortality rate of untreated penicilliosis is 100% [77]. Any delay in the initiation of antifungal therapy is associated with poor outcome whereas the therapeutic response is good with early institution of treatment [8, 52, 77]. The recommended initial treatment for penicilliosis in HIV-positive patients is intravenous amphotericin B (0.6 mg/kg) for 2 weeks followed by oral itraconazole 400 mg per day for 10 weeks [82]. Treatment with itraconazole alone has also been shown to be effective but is associated with higher relapse rate [83]. It has been recommended that itraconazole alone 400 mg/day for 8 weeks could be considered for mild disease, followed by maintenance therapy with 200 mg per day to prevent relapse [82]. Oral itraconazole is available in capsule and solution form. Oral absorption of capsule is dependent on a low gastric pH and is enhanced by food or cola beverage [84, 85]. It can be erratically absorbed in patients with AIDS patients who may have a low gastric pH and therefore serum levels should be performed if available [86]. On the other hand, itraconazole solution had a more reliable absorption with an enhanced bioavailability but has to be taken on an empty stomach [87, 88]. 

Two other important issues on the clinical management of penicilliosis in HIV-positive patients require special attention. The first is drug interaction between antifungal and antiretroviral agents. A lot of antiretrovirals are known to interact with itraconazole. Itraconazole is a substrate of CYP3A4 but can also inhibit metabolism of many CYP3A4 substrates and increased their concentration. It is known to interact with protease inhibitors, and may increase the plasma concentration of indinavir, ritonavir and saquinavir. On the other hand, indinavir and ritonavir may also increase the plasma concentration of itraconazole [89]. Nonnucleoside reverse transcriptase inhibitors (NNRTIs) significantly reduce itraconazole concentration by promoting its metabolism [90]. Maraviroc, a CCR5 antagonist, is metabolized by CYP3A4 and therefore itraconazole may increase its concentration [91]. Most nucleoside reverse transcriptase inhibitors (NRTIs) and raltegravir, an integrase inhibitor, do not have significant interactions with itraconazole. It is important to check for drug interaction before starting the antifungal or antiretroviral agents. 

The second issue is the optimal timing of initiation of HAART and the risk of development of immune restoration inflammatory syndrome (IRIS) after HAART. Penicilliosis is considered an AIDS-defining illness in endemic areas [8–10] and its diagnosis warrants initiation of HAART [92]. IRIS has only been uncommonly reported in patients with penicilliosis and usually occurred a month after the start of HAART [93–95]. Simultaneous initiation of HAART with antifungal or delayed initiation until the end of the 2 weeks of induction therapy of antifungal therapy can be considered [82]. HAART should not be withheld because of concern for possible development of IRIS. In case of severe symptomatic IRIS, a short-course of steroids may be considered [82].

## 8. Prevention

During the pre-HAART era, over half of patients developed relapse of penicilliosis within 6 months after discontinuation of antifungal treatment [83, 96]. Secondary prophylaxis with itraconazole 200 mg/day was shown to be well tolerated and highly effective with a reduction in relapse rate from 57% to 0% [96]. Therefore, it has been recommended that all patients who have completed treatment for penicilliosis should be put on secondary prophylaxis with itraconazole 200 mg/day [82]. 

With the introduction of HAART, there is growing data to suggest that secondary prophylaxis can be stopped after immune restoration [97, 98]. It is suggested that secondary prophylaxis can be stopped for patients who are receiving HAART and have a CD4 count >100/uL for over 6 months. However, secondary prophylaxis should be reintroduced if the penicilliosis relapses or the CD4 count falls below 100/uL [82].

## Figures and Tables

**Table 1:** Symptomatology of penicilliosis in HIV-positive patients.

	Study location (number of subjects)		
Signs/symptoms	Thailand [8]	India [17]	Hong Kong [18]
	(*N* = 80)	(*N* = 36)	(*N* = 47)*
Fever	93%	97%	96%
Skin lesion	71%	81%	28%
Anaemia	78%	86%	79%
Hepatomegaly	51%	39%	28%
Splenomegaly	16%		15%
Lymphadenopathy	58%	33%	62%
Diarrhoea	31%	22%	15%
Cough	49%	—	40%
Presence of other OIs	55%	77%	57%

OI: opportunistic infection.

*94% of the 47 subjects are confirmed HIV positive.

**Table 2:** Distinguishing features of microorganisms which may be confused with *Penicillium marneffei* in tissue examination [40, 56, 57].

	Similarities	Differences
*Histoplasma capsulatum*	Yeast cells with similar staining properties and size Found within histiocytes and similar inflammatory response	Budding instead of septated yeast cells

*Pneumocystis jiroveci*	Cyst form similar size stained positive silver methenamine stain	Round cysts containing single or paired comma shaped argyrophilic foci in walls

*Leishmania spp.*	Amastigotes within histiocytes in H&E section	Presence of bar shaped kinetoplasts within amastigotes seen under oil immersion, PAS stain negative

*Toxoplasma gondii*	May appear as intracellular organisms in H&E section or Giemsa stain	Size smaller, can be found within in other somatic cell types, not stained with silver methenamine

H&E: haematoxylin and eosin; PAS: periodic acid-Schiff.
